# Framing Function: Metallophthalocyanine-Based Metal–Organic Frameworks as Multifunctional Materials for Electrified Devices

**DOI:** 10.1021/accountsmr.5c00283

**Published:** 2026-01-13

**Authors:** Evan L. Cline, Hyuk-Jun Noh, Katherine A. Mirica

**Affiliations:** Department of Chemistry, 3728 Dartmouth College, Hanover, New Hampshire 03755, United States

## Abstract

Metallophthalocyanine-based metal–organic frameworks (MPc-based MOFs) have recently emerged as a class of two-dimensional (2D) materials with unique tunability for control over both structural properties and growing applications. MPc-based MOFs possess a unique set of structural characteristics due to the combination of a two-dimensional, sheet-like, porous structure and a modular, bimetallic molecularly precise chemical composition that result in emergent properties, such as electrical conductivity, modular surface chemistry, and tunable stacking properties. This combination of physical, chemical, and structural modularity has led to the promising demonstrations of MPc-based MOFs within a wide range of applications, including chemical sensing, catalysis, energy storage, and magnetoresistivity. While recent research regarding structure–property relationships of these materials has significantly advanced this field, the exploration of this class of 2D conductive MOFs has been limited by factors including the synthetic accessibility of both the functionalized MPc monomer and the crystalline framework materials, as well as the lack of structural clarity due to limitations in producing sufficiently large ordered crystals suitable for single crystal X-ray diffraction. Systematic investigation of structure–property relationships, enabled by careful control over synthetic parameters and device integration techniques, are essential for advancing the fundamental understanding and capitalizing on the applied potential of this class of materials.

This Account summarizes the development of MPc-based MOFs as a privileged class within the realm of conductive 2D framework materials. Furthermore, this Account highlights key contributions from our group, with a particular focus on how chemical modulation within MPc building blocks dictates the resulting MOF structures and their functional performance. Capitalizing on the beneficial properties of the MPc building blocks, the structural modularity of these materials provides unique access to systematic investigations of structure–property relationships. Structure–property related insights make it possible to elucidate the role of the metal within the MPc core, the bridging metal, and the heteroatomic linker on the functional performance of these materials in the context of electronically transduced chemical sensing and electrocatalysis. The multifaceted utility of this class of materials is also highlighted in both energy storage applications and magnetoresistive devices. Through a combination of iterative synthetic efforts, characterization studies, and systematic investigations into electrical devices incorporating MPc-based MOFs, this Account demonstrates that these materials are prime candidates for use in electronically transduced devices where molecular-level control can be leveraged to maximize device performance metrics. Taken together, these achievements establish MPc-based MOFs as a promising class of materials with high potential within the field of functional nanoscience.

## Graphical abstract

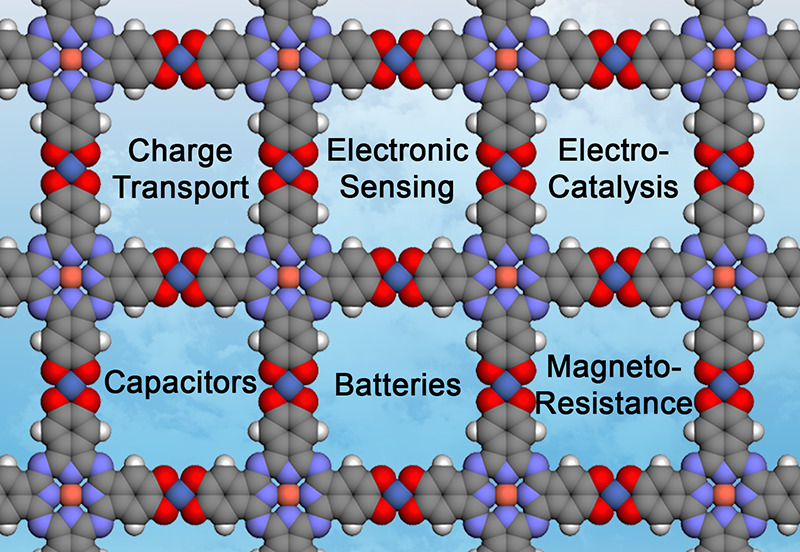

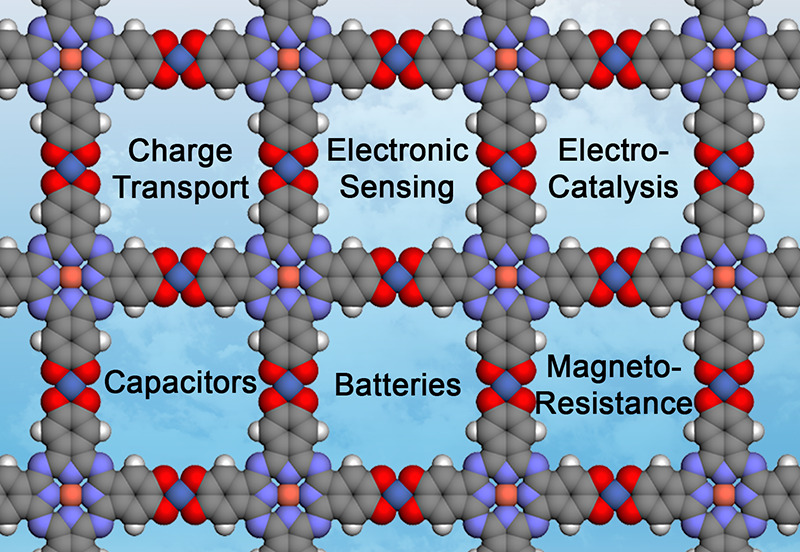

## 1 Introduction

Conductive, two-dimensional (2D) metal–organic frameworks (MOFs) are a class of atomically ordered intrinsically porous planar network structures with high chemical and structural modularity. These materials comprise of two parts: an organic linker molecule with heteroatomic linker atoms and a metal node. When properly coordinated, the integration of these components leads to structural characteristics of high periodicity, crystallinity, permanent porosity, and charge delocalization, generating a material with a high surface area and intrinsic electrical conductivity.^1^ Since the initial report of the first conductive hexahydroxytriphenylene-based 2D MOF in 2012,^2^ the field of 2D MOFs has advanced in molecular design and in material applications. While initial reports of 2D conductive MOFs utilized triphenylene and benzene based organic linkers,^3–6^ scientists have significantly expanded the structural diversity using new linker molecules (Figure 1). The strategies for this expansion have employed molecular design principles, such as extending the aromatic framework,^7,8^ installing different heteroatomic cross-linkers to enhance metal-linker d-π orbital overlap,^9,10^ and integrating heteroatoms into the linker structure itself (Table S1).^11,12^ One promising class of organic linkers in both fundamental and applied studies is that of metallophthalocyanines (MPcs) due to literature precedent indicating desirable molecular materials properties^13,14^ and a unique combination of a large, extended aromatic structure with an integrated metal atom.

**Figure 1:**
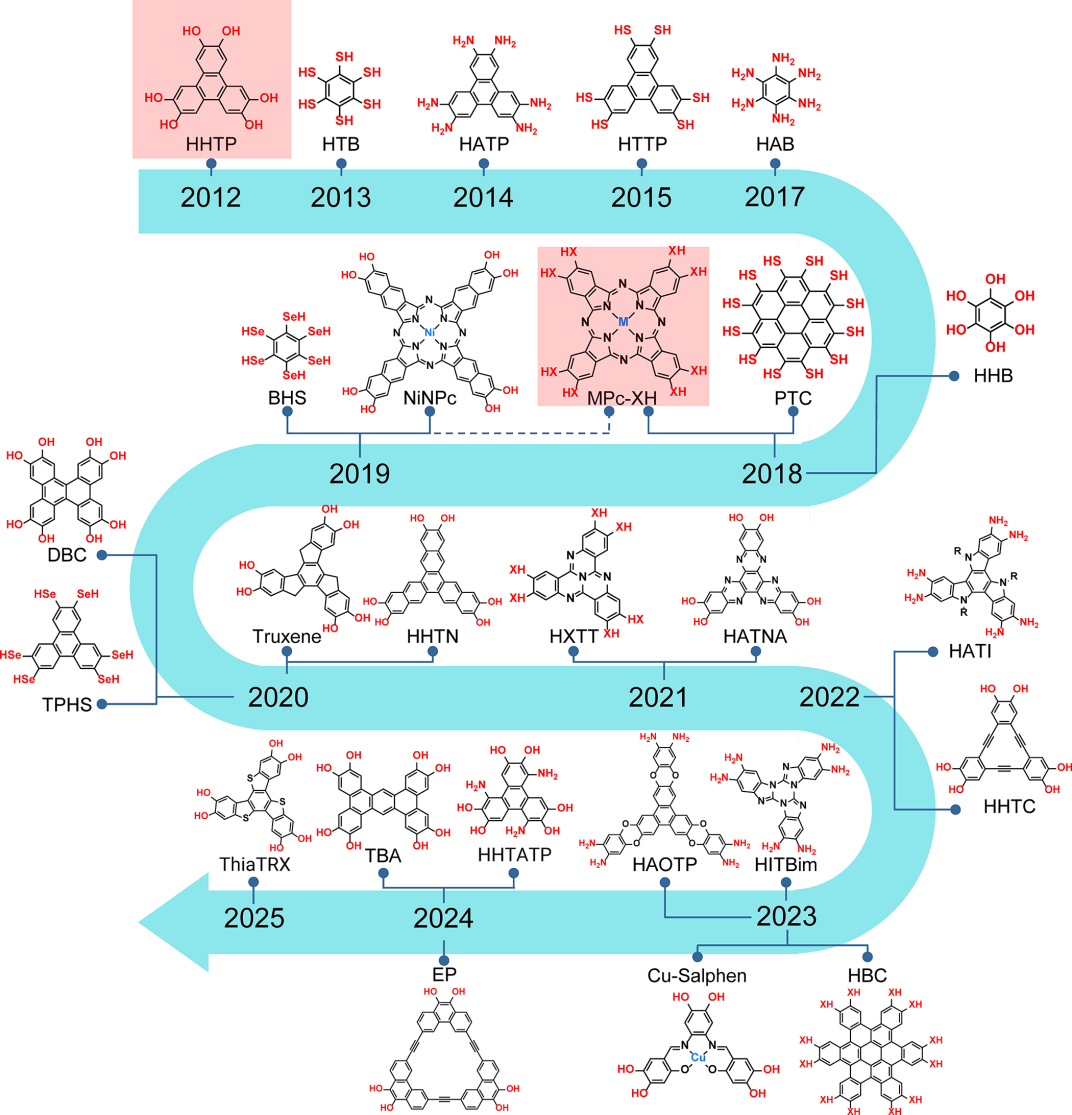
Chronological roadmap highlighting the development of 2D conductive MOFs through organic linker choice.

Building upon the initial isolation and report of MPc molecules in 1937,^15^ researchers have harnessed the structural characteristics of MPcs to form conductive frameworks with properties including a relatively low HOMO–LUMO gap,^16^ a modular magnetic moment,^17^ modifiable optical absorbance and photoconductivity,^18^ and D_4h_ framework symmetry, permitting square arrangements of the MPc molecule (Figure 2a).^19^ This unique C_4_ topological aspect of MPc monomers relative to other 2D MOF organic linkers engenders MPc molecules with the potential to form frameworks with Lieb lattices.^19,20^ Lieb lattices are 2D, edge-depleted square lattices with Dirac-flat band structures, which can produce interesting quantum states such as superconductivity and ground state ferromagnetism.^19,20^ However, reports of designed materials exhibiting Lieb lattice architectures remain limited.

**Figure 2:**
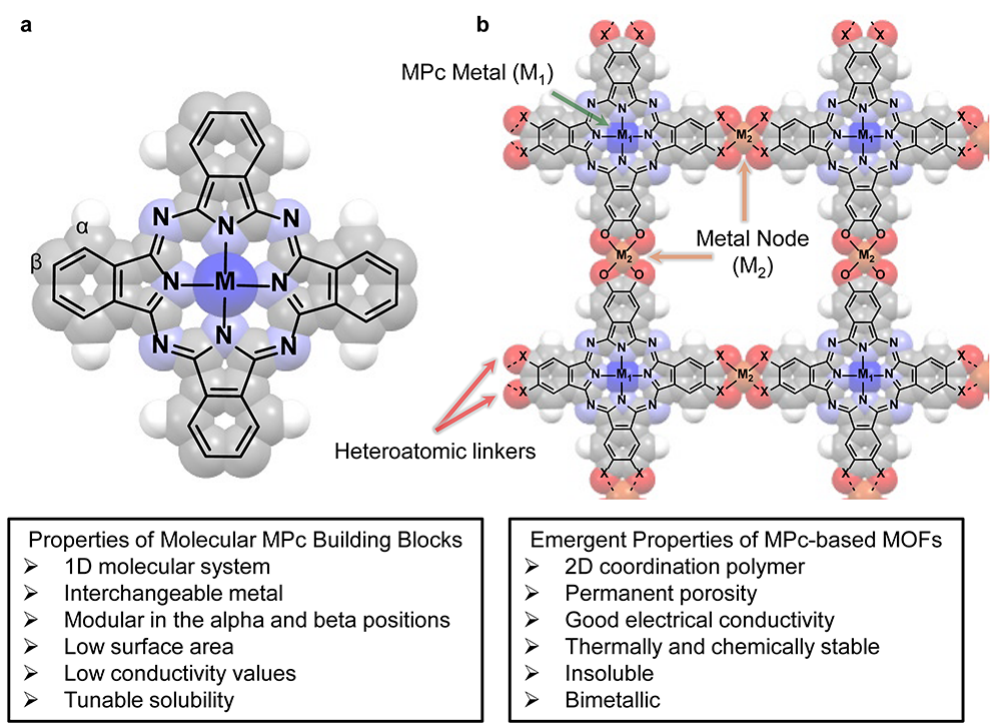
(a) MPc molecular structure demonstrating C_4_ symmetry. (b) Modular example of an MPc-based MOF. Examples of properties of both MPc and MPc-based MOFs.

The first report of a MOF synthesized from MPc ligands in 2018 by Jia et al. (herein referred to as MPc-based MOFs)^21^ both showcased how to construct highly modular MOFs within this class of materials and demonstrated the potential of MPc-based MOFs in electronic applications (Figure 2b). Moving forward, expanding on this design has showcased the unique chemical modularity of MPc-based MOFs, originating from the two tunable metal atoms, one in the phthalocyanine core (M_1_) and one at the metal node position (M_2_), as well as variable heteroatomic cross-linker atoms (XH), resulting in a general MOF structure of M_1_Pc-XH-M_2_. This class of materials features emergent functionality with tunable properties, such as bimetallicity, high surface areas,^22^ electrical conductivity with *p*-type semiconducting behavior,^23,24^ good thermal stability and relative chemical stability under toxic gases and electrochemical environments,^8,25–27^ and iterant magnetism^24^ (Table 1). Researchers have gained insight into the core structure–property relationships governing MPc-based MOFs, building upon these relationships to showcase utility in applications such as transistors,^28^ chemical sensing,^8,25^ catalysis,^27^ energy storage,^29–31^ and spintronics.^31^ Despite recent reporting of MPc-based MOFs, which includes a wide range of molecular iterations to the MPc-XH-M framework structures (Table 1), the implicit connection between chemical modulations on the framework and emergent structure–function–performance properties of the MOF remains largely unknown. Thus, investigations into the cause and effect of chemical modulations within the framework components (MPc organic linker and metal node) on the structure–property relationships and the application-specific performance of the MOF are imperative to advance the field of conductive 2D MOFs.

**Table 1:** Summary of Reported MPc-Based MOFs Including Details of M_1_, M_2_, and Heteroatomic Cross-Linker, Electrical Conductivity, Surface Area, and Reported Material Application with *Vide Infra* Corresponding Section^a^

M_1_ Metal	Cross-linker	M_2_ Metal	Reported Conductivity (S/m)	BET Surface Area (m^2^/g)	*Vide infra* section; Application
**Cu**	**O**	**Cu**	0.016 × 10^–6^ (2 point [2-pt] probe),^30^ 5.0 (EIS),^34^ 0.44 (4-pt probe),^31^ 9.3 (4-pt probe),^23^ and 0.02 × 10^–3^ (2-pt probe on film)^35^	358,^30^	3.3; Energy Storage,^30^
				264,^31^	3.2; Electrocatalysis,^34,36,37^
				448 ^23^	2.2; Charge Transport,^23,28^
					3.4; Magnetism^31^
		Co*	n/a	412	3.2; Electrocatalysis^37^
		**Zn**	0.69 (I_2_ doped, EIS),^38^ 2.9 (4-pt probe)^23^	378,^36^	3.2; Electrocatalysis,^36^
				460 ^23^	2.2; Charge Transport^23^
		Fe*	0.97 (I_2_ doped, EIS)	n/a	3.2; Electrocatalysis^38^
		**Ni**	0.97 (I_2_ doped, EIS),^38^	477 ^23^	3.2; Electrocatalysis,^38^
			4.6 (4-pt probe)^23^		2.2; Charge Transport^23^
	**NH**_**2**_	**Ni**	0.010 (Van der Pauw),^22^ 0.80 (4-pt probe),^39^ −9, 0.060 (2-pt probe),^40^	690,^22^	3.3; Energy Storage,^22^
			0.068 (4-pt probe)^41^	602,^39^	3.1; Chemical Sensing,^40^
				455 ^41^	2.2; Charge Transport^41^
		**Cu**	6.0 × 10^–4^ (4-pt probe),^39^	556,^39^	3.3; Energy Storage,^39^
			0.013 (4-pt probe)^41^	455 ^41^	2.2; Charge Transport^41^
		**Co**	0.035 (4-pt probe)	183	2.2; Charge Transport^41^
	**S**	**Ni**	1.0 × 10^–4^ (van der Pauw)	219	3.3; Energy Storage^29^
**Ni**	**O**	**Ni**	0.72 × 10^–4^ (4-pt probe),^8^	101,^8^	3.1; Chemical Sensing,^8^
			4.8 × 10^–5^ (2-pt probe)^42^	180 ^42^	3.2; Electrocatalysis^42^
		**Cu**	1.43 × 10^–4^ (4-pt probe),^8^	284,^8^ 486,^25^ 264,^31^	3.1; Chemical Sensing,^8^
			1.0 × 10^–5^ (4-pt probe),^25^ 6.6 × 10^–2^ (4-pt probe),^31^ 1.04 × 10^–2^ (4-pt probe)^27^	421 ^27^	3.2; Electrocatalysis,^27^
					3.4; Magnetism,^31^
		**Co**	1.1 × 10^–6^ (4-pt probe)	193 ^43^	3.2 Electrocatalysis^43^
		Zn*	n/a	n/a	3.2; Electrocatalysis^44^
	**NH**_**2**_	**Ni**	2.0 × 10^–2^ (4-pt probe, thin film),^21^ 3.0 × 10^–3^ (2-pt probe),^40^ 5.43 (4-pt probe),^41^ 2.73 × 10^–4^ (4-pt probe)^27^	593,^40^	3.2; Electrocatalysis,^21,27^
				412,^41^	2.2; Charge Transport,^41^
				628 ^27^	3.1; Chemical Sensing,^40^
		**CoTAA**	8.16 × 10^–3^ (4-pt probe)	186 ^12^	3.1; Chemical Sensing^12^
		**Co**	1.0 × 10^–2^ (4-pt probe)	414 ^41^	2.2; Charge Transport^41^
		**Cu**	1.73 (4-pt probe)	314	2.2; Charge Transport^41^
	**S**	**Co**	1.7 x10^–7^ (4-pt probe)	n/a	3.2; Electrocatalysis^26^
		**Ni**	4.6 × 10^–2^ (4-pt probe)	n/a	3.2; Electrocatalysis^26^
		**Cu**	1.5 × 10^–6^ (4-pt probe)	n/a	3.2; Electrocatalysis^26^
**Fe**	**O**	**Fe**	2.0 × 10^–3^ (4-pt probe)	206 ^24^	3.4; Magnetism^24^
		Cu*	n/a	n/a	2.2; Charge Transport^28^
**Co**	**O**	**Cu**	3.4 × 10^–5^ (4-pt probe),^25^	411,^25^	3.1; Chemical Sensing,^25^
			2.12 × 10^–3^ (4-pt probe)^27^	582 ^27^	3.2; Electrocatalysis,^27^
		Mn*	n/a	n/a	3.3; Energy Storage^45^
	**NH**_**2**_	**Ni**	2.1 × 10^–2^ (4-pt probe)	454	2.2; Charge Transport^41^
		**Cu**	5.79 × 10^–3^ (4-pt probe)^27^	349,^27^	3.2; Electrocatalysis,^27^
			2.0 × 10^–2^ (4-pt probe),^41^	548 ^41^	2.2; Charge Transport^41^
		**Co**	8.9 × 10^–5^ (4-pt probe)	119	2.2; Charge Transport^41^
**Zn**	**O**	Ni*	n/a	n/a	3.2; Electrocatalysis^42^
		Zn*	n/a	n/a	3.2; Electrocatalysis^36^
		Cu*	n/a	n/a	3.2; Electrocatalysis^36^
**H**_**2**_	**O**	**Cu**	3.72 × 10^–7^ (4-pt probe)^27^	364,^27^	3.2; Electrocatalysis,^27^
			8.4 × 10^–2^(4-pt probe)^31^	292 ^31^	3.4; Magnetism^31^
	**NH**_**2**_	**Cu**	5.32 × 10^–5^ (4-pt probe)	181	3.2; Electrocatalysis^27^

a Asterisks indicate partial characterization included.

Previous reviews detailed the impact of MPc molecular engineering toward emergent function,^18^ while Accounts have discussed the status of 2D MOF research^32^ and MPc-based MOFs used within specific applications, such as chemiresistive sensing.^33^ Building upon these prior efforts, this Account summarizes advances in MPc-based MOFs, focusing on how chemical modularity governs structure–property relationships. We illustrate how fundamental structure–property insights translate into the performance of MPc-based MOFs in electronic devices, showcasing their potential across diverse applications. Highlighting this progression from structural control to device-level functionality underscores the importance of MPc-based MOFs as versatile platforms for bridging practical technologies with fundamental chemistry. Sections pair emphasis on structure–property relationships and application-specific studies with exceptional performance of MPc-based MOFs within chemical sensing, catalysis, energy storage and magnetism. We highlight the chemical modularity exhibited for MPc-based MOF reports, detailing the reported analogs of MPc-based MOFs to date. This Account showcases the potential of MPc-based MOFs, discusses current challenges facing the field, and outlines future research directions for advancing the field.

## 2 Structure–Property Relationships of MPc-Based MOFs

### 2.1 Impact of the M_1_ Metal on the Structure–Property Relationships

Harnessing the modularity of the phthalocyanine organic monomer (M_1_ metal location), studies have reported the synthesis of MOFs with MPcs containing first-row transition metals from Fe to Zn (Table 1). A critical first step for MPc-based MOF structure–property studies is access to pure MPc monomer to ensure the coordination-driven assembly of the monomer into crystalline MOF domains. Currently, the predominant strategy to obtain pure MPc monomer with heteroatomic linkers installed include the tetramerization and eventual deprotection of functionalized phthalonitriles.^8,23,25,27^ While this strategy has proven to be successful at the laboratory scale, drawbacks including Soxhlet extractions of phthalonitrile material,^23^ strict temperature control over the tetramerization into the phthalocyanine to inhibit metal nanoparticle formation,^46^ and the potential for inseparable impurities within the MPc monomer structure^18^ enhance the difficulty of synthesizing MOF-grade MPc monomeric material. Even with highly pure MPc monomers, the quality of the resulting MPc-based MOFs relies on precise control of synthetic parameters, including solvent composition, basic modulators, reaction temperature, and reaction time.^8,23^ Typically, MPc monomers are soluble in aprotic solvents such as *N,N*-dimethylformamide and dimethyl sulfoxide. After dissolving the monomers in the solvent, water can be added to modulate the reaction mixture solubility. A base, such as ammonium hydroxide, can then be introduced to deprotonate the heteroatomic linkers of the MPc during the reaction. Finally, the reaction temperature and time are iteratively refined to identify the optimal conditions for MOF crystal growth.

The identity of the M_1_ metal greatly impacts the physicochemical properties of the MOF material. While researchers have noted the impact of the M_1_ metal on MOF performance in applications,^8,25,27^ the corresponding influence on the emergent structure–property relationships of the MOFs remains insufficiently studied. Comparing the effect of the metal atom in both the M_1_ (MPc) and M_2_ (metal node) location, Chen et al. determined that the M_1_ metal exerted a dominant influence over the optical band gap and overall electronic structure of the framework.^41^ Using density functional theory (DFT) calculations, the authors established that among the nine MPc-based MOFs with Co, Ni, and Cu as the M_1_ and M_2_ metals, the M_1_ metal had a greater impact on the band gap, specifically impacting the energy level of the LUMO.^41^ The average experimental LUMO levels of −4.05 eV, −4.25 eV, and −4.16 eV were found to Co, Cu and Ni analogs, respectively, while the HOMO levels remained indistinguishable.^41^

### 2.2 Impact of the M_2_ Metal on the Structure–Property Relationships

Because of the higher relative content of M_2_ to M_1_ metals within the MPc-based MOF unit cell and the relative ease of synthetic control over the M_2_ metal of the MOF compared to the M_1_ metal, delineating the influence of the M_2_ metal atom on the structure–property relationships of the MOFs is more well studied. For instance, Bao and co-workers found that the identity of the M_2_ metal node correlated with the rate of MOF formation, with MOFs linked with Ni forming slower relative to Co and Cu analogs, according to a UV–vis study.^41^ This finding suggested that the rate of MOF formation reaction may be inversely related to the crystallinity of the MOF.^41^

This link between the impact of M_2_ metal on the formation and crystallinity of the MOF has implications for the emergent structure–property relationships. As direct studies for comparing materials with different M_2_ linking metal nodes for MPc-based MOFs can prove difficult due to inherent differences in material crystallinity, researchers have employed various strategies to ensure fair comparisons of materials. In contrast to the study by Bao and co-workers, which affixed the MOF formation reaction conditions for all materials within the study,^41^ a study employed by our group in 2025 optimized the MOF reaction conditions to maximize the crystallinity for each respective material.^23^ We observed that copperphthalocyanine (CuPc) MOFs with Zn metal nodes (M_2_) showed the highest crystallinity relative to CuPc-O-Ni and CuPc-O-Cu and the largest crystallite grains for MPc-based MOFs reported to date (Figure 3).^23^ Furthermore, high-resolution transmission electron microscopy (HR-TEM) analysis of CuPc-O-Zn (Figure 3f-h) revealed that, unlike MPc-based MOFs linked with Fe, Co, Ni, or Cu which stack in an AA-eclipsed interlayer stacking pattern, MPc-based MOFs with Zn metal nodes stacked in an AA-inclined stacking pattern.^23^ This inclined stacking pattern of CuPc-O-Zn led to less orbital overlap in the “through-space” charge transport and thus a lower 4-pt probe conductivity, compared to CuPc-O-Ni and CuPc-O-Cu (Figure 3j and k). This finding for CuPc-O-Zn contradicted the typical direct relationship between crystallinity, crystal size, and material conductivity for previously reported, analogous iterations of MPc-based MOFs.^8,23,27,41^ We rationalized this discrepancy in crystallite size and conductivity based on the possibility that the dominant charge transport pathway for MPc-based MOFs is the “through-space” pathway, which may be hindered by the inclined stacking.

**Figure 3:**
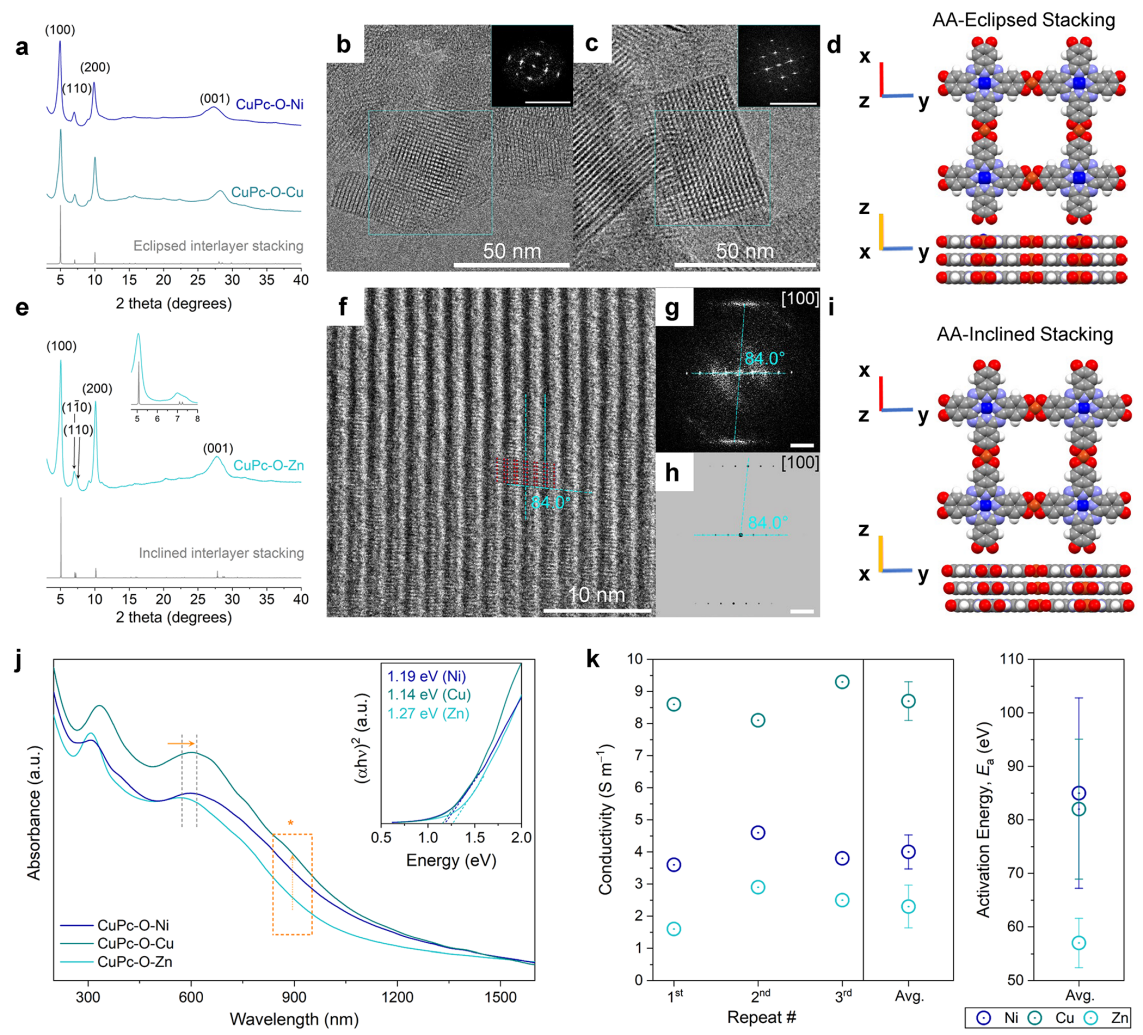
(a) PXRD patterns of CuPc-O-Ni and CuPc-O-Cu. HR-TEM images of (b) CuPc-O-Ni, and (c) CuPc-O-Cu. Insets are fast Fourier transform (FFT) images. Scale bars: 2 nm^−1^. (d) Simulated AA-eclipsed stacking pattern. (e) PXRD pattern of CuPc-O-Zn. HR-TEM image of (f) CuPc-O-Zn. (g) Experimental and (h) simulated FFT images. Scale bars: 1 nm^−1^. (i) Simulated AA-inclined stacking pattern. (j) UV−vis-NIR spectra for CuPc-O-M MOFs. Insets: corresponding normalized Tauc plots, and (k) the electrical conductivities of CuPc-O-Ni (navy), CuPc−O-Cu (teal), and CuPc-O-Zn (cyan) as a function of temperature (from 180 to 300 K) measured by 4-pt probe method. Right: temperature dependence of the electrical conductivities of CuPc-O-M MOFs plotted according to the Arrhenius equation. Reproduced with permission from reference 23. Copyright 2025 American Chemical Society.

### 2.3 The Role of Heteroatomic Linkers on the Structure–Property Relationships

Studies suggest that the heteroatomic linker may also influence structure–property relationships of MPc-based MOFs. A study from our group found that within a group of MPc-based MOFs with metal bis­(diimine) and metal (bis)­dioxolene heteroatomic linkers, the cross-linker is less dominant than the M_1_ metal for the electrocatalytic reduction of CO_2_ to CO.^27^ However, in the same study, we showed that the heteroatomic cross-linker influenced the redox activity, with the CoPc-Cu-O and NiPc-Cu-O MOFs (containing Cu bis­(dioxolene) moieties), demonstrating larger redox peak currents in cyclic voltammetry (CV) and thus a higher number of electroactive sites versus CoPc-NH-Cu and NiPc-NH-Cu MOFs (containing Cu bis­(diimine) moieties).^27^ In the context of magnetism, a computational study by Li et al. showed that MPc-based MOFs with Ni bis­(diimine) linkages demonstrated the higher planarity, relative to Ni bis­(dioxolene) and Ni bis­(dithiolene) linkages.^47^ This improvement in planarity indicated greater orbital overlap between the Ni bis­(diimine) linkages and the Ni M_2_ node, facilitating efficient magnetic exchange and π-electron delocalization through the framework.^47^ To date, the theorized improvement in orbital overlap and π electron delocalization within Ni bis­(diimine) containing MPc-based MOFs has not yet been experimentally investigated from a structure–property relationships perspective.

While studies on MOFs with metal bis­(dioxolene) and metal bis­(diimine) linkages have provided valuable insights into their fundamental structural and electronic properties, investigations on metal bis­(dithiolene)-linked MOFs remain limited. Although Feng and co-workers reported MOFs with metal bis­(dithiolene) moieties,^29^ this investigation focused on energy storage performance, leaving their fundamental structure–property relationships largely unexplored. Taken together, these findings suggest that the heteroatomic cross-linker mainly functions by tuning the in-plane orbital overlap with the M_2_ metal node.

## 3 Applications of MPc-Based MOFs

### 3.1 Chemiresistive Sensing

One promising application for MPc-based MOFs is chemiresistive detection due to the properties of electrical conductivity, large surface area, low dimensionality, and combined modular surface chemistry within the bimetallic framework structure. The combination of these features enable MPc-based MOFs to perform as senstive chemiresistive materials with tailorable surface chemistries for targeted host–guest interactions. In 2019, we reported the first MPc-based MOFs for chemiresistive sensing, NiPc-O-Cu and NiPc-O-Ni, demonstrating limits of detection (LODs) toward NO, H_2_S, and NH_3_ of 1.0, 19, and 310 parts-per-billion (ppb), respectively (Figure 4).^8^ Spectroscopic investigations using XPS and EPR revealed that the chemiresistive sensor response was induced by charge transfer interactions resulting from gaseous molecules interacting with the MOF.^8^ We demonstrated that tuning the M_2_ metal node and extending the pore structure of the MPc-based MOF materials are promising strategies for achieving differentiation of gaseous analytes using principal component analysis (PCA).^8^

**Figure 4:**
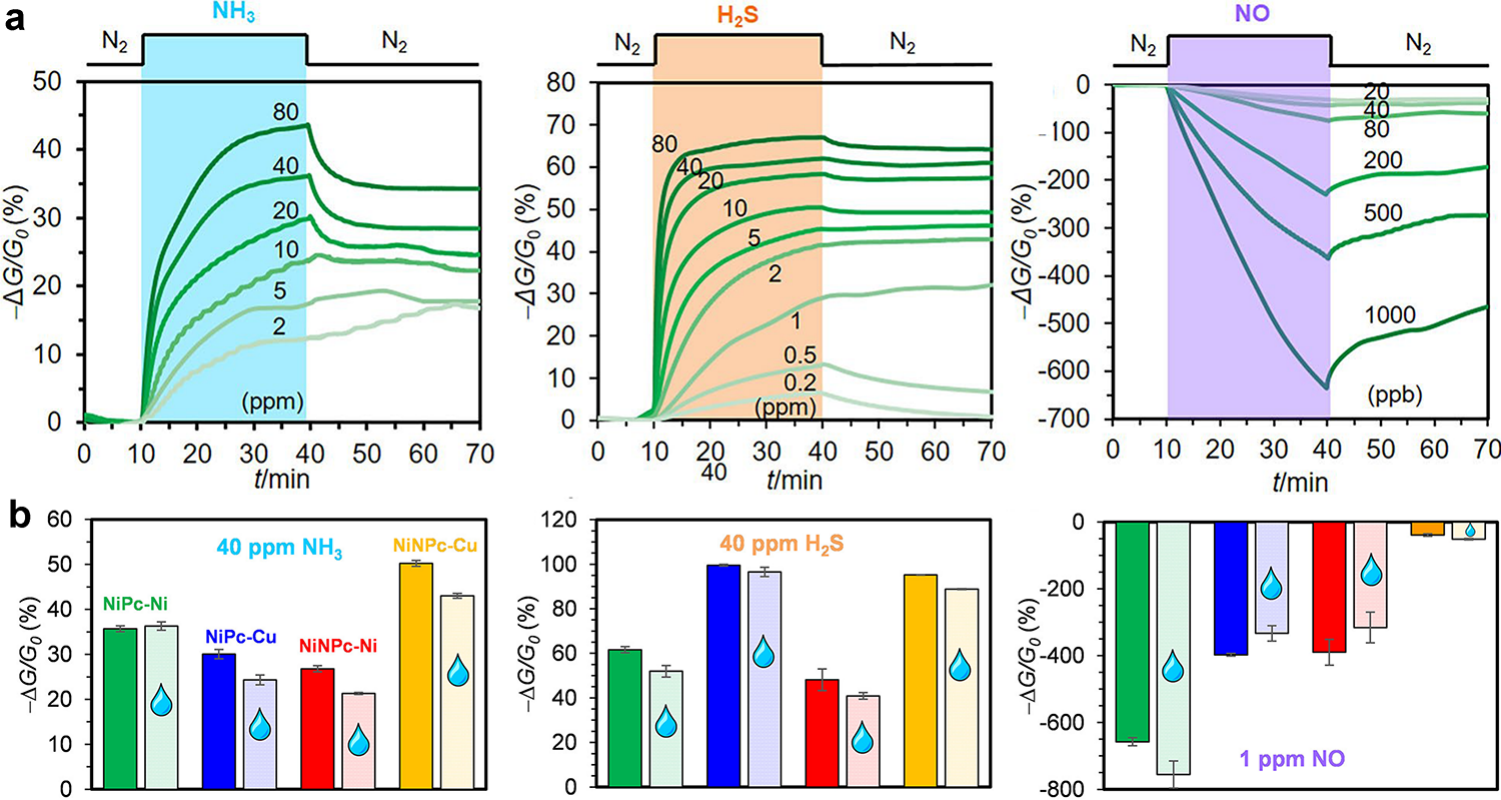
(a) Chemiresistive sensing traces of NiPc-O-Ni toward concentrations of NH_3_, H_2_S, and NO. (b) Sensing responses of NiPc-O-M and NiNPc-O-M MOFs upon 30 min exposure to 40 ppm (ppm) of NH_3_, 40 ppm of H_2_S, and 1 ppm of NO. Reproduced with permission from reference 8. Copyright 2019 American Chemical Society.

Leveraging the structural modularity of MPc-based MOFs is a reliable approach to enhance both the sensitivity and selectivity of MPc-based MOFs. Huang and co-workers reported a novel MOF-based chemiresistor, which integrated Co­(II)-tetraaza[14]­annulene (CoTAA) linkages to form NiPc-CoTAA.^12^ Using this unique MOF structure, the authors fabricated thin film chemiresistors *via* a steam-assisted conversion approach to realize sensitive NO_2_ detection. The authors theorized that the heightened sensitivity of NiPc-CoTAA toward NO_2_, relative to that of NO, NH_3_, H_2_S, and H_2_, resulted from an intermolecular hydrogen-bonding interaction with nitrogen atoms in the MOF framework.^12^ In a separate study, Dong and co-workers modified the surfaces of CuPc-NH-Ni and NiPc-NH-Ni with various insulating silanes, including (3-aminopropyl)­trimethoxysilane (APTMS), phenyltrichlorosilane (PTCS), and octadecyltrimethoxysilane (OTMS) to investigate the impact of induced hydrophobicity on chemiresistive sensitivity.^40^ By grafting MPc-based MOFs with silanes, the authors demonstrated rapid and highly reversible sensor responses toward volatile, polar analytes including methanol, ethanol, and water.^40^

While the chemiresistive sensitivity of MPc-based MOFs is well established, researchers continue to investigate the host–guest chemistry that governs the sensor response. In another study from our group, we systematically compared CoPc-O-Cu and NiPc-O-Cu in the context of the first chemiresistive detection of carbon monoxide (CO) using a conductive MOF, ultimately achieving an LOD of 530 ppb (Figure 5).^25^ We observed that CoPc-O-Cu reversibly detected CO in the concentration range of 10–80 parts-per-million (ppm) over multiple exposures and under humidified air (5000 ppm of H_2_O) (Figure 5b). Diffuse reflectance infrared Fourier transform spectroscopy (DRIFTS) provided insight into molecular-level interactions, revealing that CO binds more strongly to the Cu metal node within CoPc-O-Cu than NiPc-O-Cu due to electronic tuning of the phthalocyanine structure by the Co M_1_ metal (Figure 5c-e).^25^ This finding demonstrated the impact of structural tuning on the chemiresistive sensitivity of MPc-based MOFs. In summary, MOF-based chemiresistors show promise in achieving highly sensitive chemical detection at room temperature and with low driving voltages. Fully harnessing the high modularity and low dimensionality of MPc-based MOF materials remain a promising strategy for strategic tuning of sensitivity and selectivity within chemiresistive devices.

**Figure 5:**
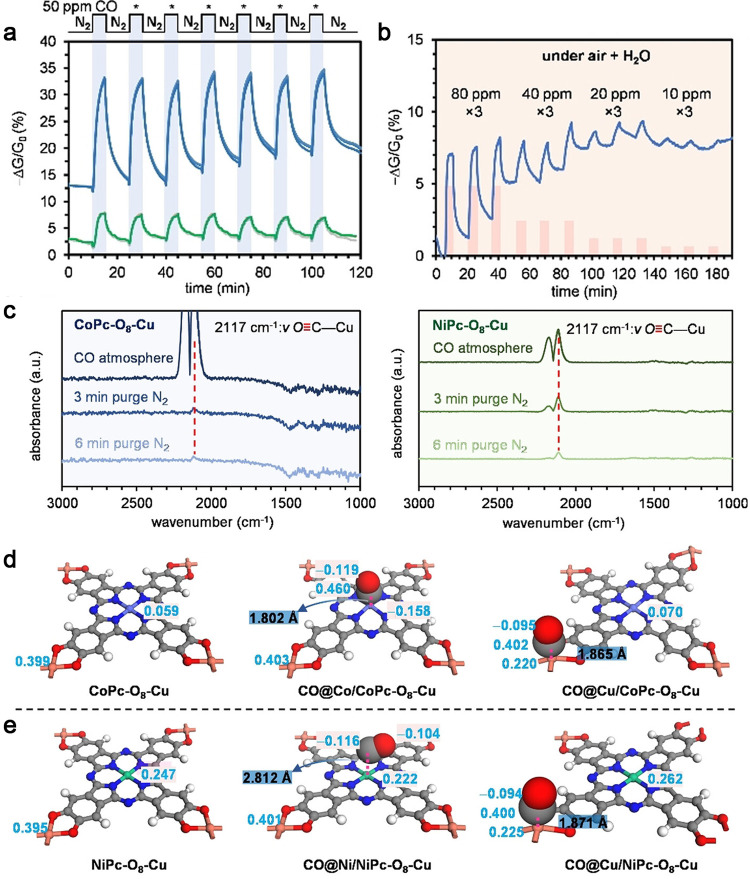
(a) Sensing traces of 7 sequential exposure-recovery cycles to 50 ppm of CO using CoPc-O-Cu (blue) and NiPc-O-Cu (green). Each cycle comprised a 5 min exposure and 10 min recovery. (b) Sensing traces of CoPc-O-Cu to consecutive exposure-recovery cycles to 80, 40, 20, and 10 ppm of CO in the air with 5000 ppm of H_2_O. (c) DRIFTS spectra of CoPc-O-Cu (left) and NiPc-O-Cu (right) after exposure to 1% CO (10 000 ppm) for 6 min. The spectra are presented as double-beam experiments with pristine MPc-O-Cu MOFs used as the reference. (d) The optimized structures of CoPc-O-Cu, CO@Co/CoPc-O-Cu, and CO@Cu/CoPc-O-Cu. (e) Optimized structures of NiPc-O-Cu, CO@Ni/NiPc-O-Cu, and CO@Cu/NiPc-O-Cu. The calculated values of the Mulliken charge are labeled with blue. The CO···M lengths are labeled with black. Reproduced with permission from reference 25. Copyright 2022 Wiley-VCH.

### 3.2 Electrocatalysts

MPc-based MOFs are emerging as promising platforms for electrocatalysis due to their permanent porosity, intrinsic electrical conductivity, and modular surface chemistry. Their tunable structures facilitate the construction of programmable catalytic active sites, that control selectivity characterized by Faradaic efficiency, while intrinsic electrical conductivity can maximize reaction kinetics and the resulting current density. Our group revealed that the catalytic performance of MPc-based MOFs in the carbon dioxide reduction reaction (CO_2_RR) can be controlled by the choice of two structural features: (1) the metal within the MPc catalytic subunit (M = Co vs Ni) and (2) the identity of the heteroatomic cross-linkers (X = O vs NH) (Figure 6).^27^ Specifically, CoPc-based metal bis­(dioxolene)-linked MOFs exhibited lower activation energies for the formation of carboxyl (*COOH) intermediates, which may account for their enhanced selectivity toward CO production.^27^ These findings demonstrated that the electrocatalytic activity of MPc-based MOFs can be directly modulated by the structure of the MOF, while the intrinsic conductivity of the MOF can promote good current density without added conductive fillers (−9.5 mA cm^–2^).

**Figure 6:**
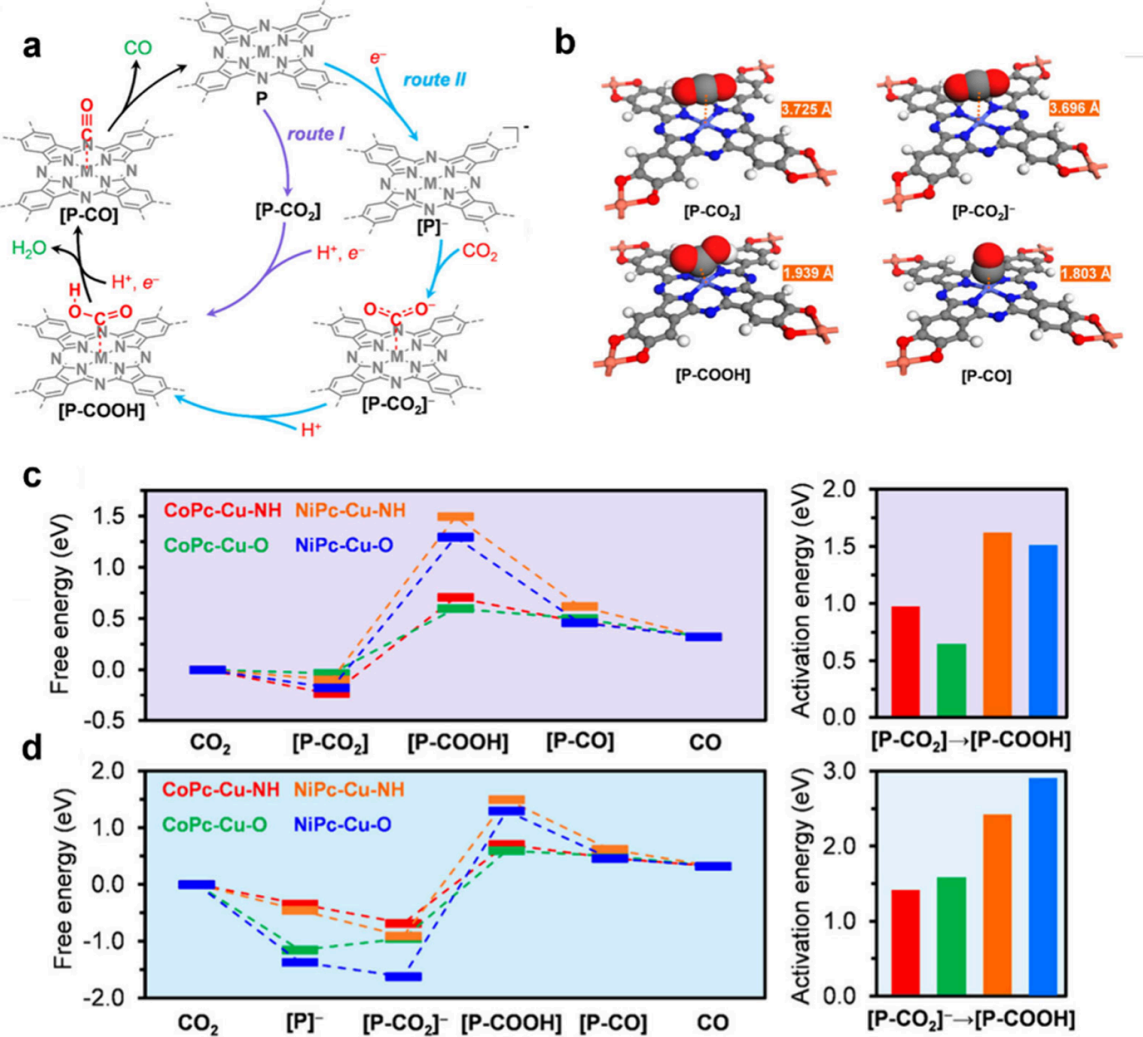
(a) Proposed catalytic mechanism for electrochemical reduction of CO_2_ to CO by MPc sites of MPc-XH-Cu MOFs, which contains two reaction pathways. (b) Structures of the catalyst and key reaction intermediates of the proposed reaction mechanism for CoPc-O-Cu mediated reaction. (c, d) Free energy profiles for electrochemical reduction of CO_2_ to CO catalyzed by CoPc-NH-Cu (red), CoPc-O-Cu (green), NiPc-NH-Cu (orange), and NiPc-O-Cu (blue) under the standard condition and electrode potential of 0 V (vs standard hydrogen electrode) through the reaction pathway of (c) route I and (d) route II. The activation energy values are plotted on the right side as bar graphs and color-coded for the four MOFs correspondingly. In route I and II, the steps with the highest activation energy values are [P-CO_2_]→[P–COOH] and [P–CO_2_]^−^→[P–COOH], respectively. Reproduced with permission from reference 27. Copyright 2020 American Chemical Society.

Within these insights into the impact of structural modularity on MPc-based MOF catalytic performance, researchers have expanded the utility of MPc-based MOFs in catalytic reduction reactions. For deeper insights into CO_2_RR conversion, Chen and co-workers developed CuPc-based MOFs with Cu bis­(dioxolene) linkages (CuPc-O-Cu) as the electrocatalyst for the reduction of CO_2_ to C_2_H_4._ ^34^ These MOFs achieved a Faradaic efficiency (FE) of 50% and a current density of 7.3 mA cm^–2^, highlighting the synergistic effect between the CuPc unit (M_1_) and the CuO_4_ unit (M_2_) in facilitating C–C coupling.^34^ In a separate study, Feng and co-workers reported a CuPc-based 2D MOF featuring Co bis­(dioxolene) linkages (CuPc-O-Co), which exhibited high electrocatalytic oxygen reduction reaction (ORR) activity in alkaline media (*E*_1/2_ = 0.83 V vs RHE, *n* = 3.93, and *j*_L_ = 5.3 mA cm^–2^).^37^ Mechanistic investigations using *in situ* Raman spectro-electrochemistry and theoretical modeling revealed that Co metal nodes (M_2_ position) served as the active sites for ORR. Furthermore, CuPc-O-Cu demonstrated excellent performance as a cathodic electrocatalyst in Zn-air batteries, outperforming benchmark Pt/C electrocatalysts.

MPc-based MOFs have also been investigated for electrocatalytic oxidation reactions, demonstrating the versatility of MPc-based MOFs as electrocatalysts. In 2021, Song and co-workers designed and synthesized a series of MPc-based MOFs by varying both the MPc center (M_1_ = Ni or Zn) and the M_2_ metal node (M_2_ = Ni or Zn).^44^ Electrochemical measurements combined with d-band center calculations revealed that NiPc-O-Ni exhibits the highest oxygen evolution reaction (OER) activity, indicating that both Ni–N_4_ (M_1_ position) and Ni–O_4_ sites (M_2_ position) synergistically contribute to the catalytic performance for the OER. Recently, Dong and co-workers extended the electrocatalytic scope of MPc-based MOFs to the electrocatalytic glycerol oxidation reaction (GOR).^26^ In their work, octathiolphthalocyaninato nickel (NiPc­(SH)_8_) was coordinated with various M_2_ metal nodes to form a series of MOFs denoted as M_2_[NiPcS_8_] (M = Co/Ni/Cu). Among these MOFs, Co_2_[NiPcS_8_] supported on carbon paper exhibited superior GOR performance, achieving a low potential of 1.35 V vs RHE at 10 mA cm^–2^. The enhanced activity of Co_2_[NiPcS_8_], compared to its Ni- and Cu-based analogues, is attributed to the fast kinetics and high activity of CoS_4_ sites (M_2_) for GOR.

In summary, a wide range of conductive MPc-based MOFs have exhibited promising performance as electrocatalysts, demonstrating that both the heteroatomic linkers (X = O, NH, S) and the variable permutations of M_1_ and M_2_ metal combinations play critical roles in tuning electrocatalytic performance through the modulation of their electronic structures and molecular affinities. These structural parameters synergistically influence the electronic properties of the framework, active site availability, and reaction kinetics, offering a versatile platform for the strategic, rational design of high-performance electrocatalysts. Future studies may look to probe the limits of both the thermal stability and the chemical stability of MPc-based MOFs with both guest molecules and electrolyte species post catalysis.

### 3.3 Energy Storage Applications

Electrified investigations into MPc-based MOFs have revealed their potential within energy storage applications, including capacitors and batteries, capitalizing on their modular structural properties and the high density of redox-active sites within the framework. Feng and co-workers developed Ni_2_[CuPc­(NH)_8_]/graphene hybrids for capacitor devices.^39^ These capacitors delivered an areal capacitance of 18.9 mF cm^–2^ along with excellent cycling stability, retaining 91.4% of capacitance retention after 5000 charge/discharge cycles. Combining this electrochemical investigation with DFT calculations revealed that continuous multielectron Faradaic reactions occurred at the redox-active M–N_4_ linkages (M_2_ site, cation storage) and CuPc building blocks (M_1_ site, anion storage), underscoring the structural advantages of MPc-based MOFs for capacitors (Figure 7).^39^ In an effort to expand the operating voltage windows and temperature range of MPc-based MOF capacitors by utilizing nonaqueous electrolytes, Feng and co-workers recently developed a novel Ni_2_[CuPcS_8_] MOF, wherein CuPc is linked by Ni bis­(dithiolene) (Ni−S4).^29^ This Ni_2_[CuPcS_8_] MOF displayed outstanding pseudocapacitive properties in a nonaqueous electrolyte (1 M TEABF_4_/acetonitrile), delivering a superior specific capacitance (312 F g^–1^) and remarkable cycling stability (93.5% after 10,000 cycles).^29^

**Figure 7:**
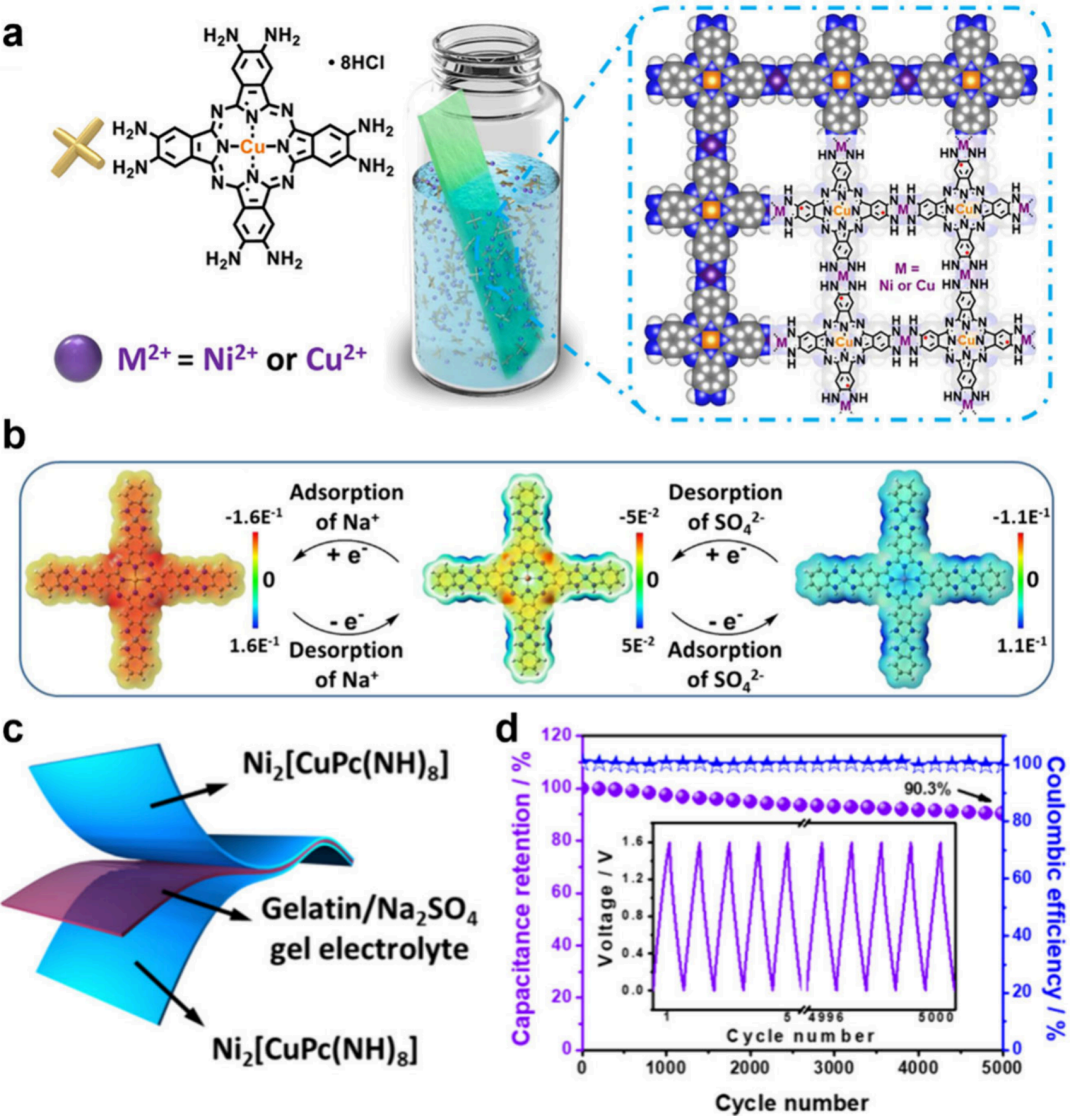
(a) Schematic illustration of the *in situ* growth of CuPc-NH-M on carbon cloth. (b) Simulated molecular electrostatic potential electronic states of CuPc-NH-Ni during the charge/discharge process. (c) Illustration of a symmetric supercapacitor based on two CuPc-NH-Ni electrodes, and (d) cycling stability and Coulombic efficiency of CuPc-NH-Ni SSCs at 10 A g^–1^. The inset shows the first five and the last five GCD curves of CuPc-NH-Ni-SSCs. Reproduced with permission from reference 39. Copyright 2021 American Chemical Society.

Within the field of energy storage applications, MPc-based MOFs have also shown promise within a range of diverse battery applications, including lithium-ion batteries (LIBs), sodium–iodine (Na–I_2_) batteries, and lithium–carbon dioxide (Li–CO_2_) batteries. For instance, Kimizuka and co-workers reported CuPc-O-Cu which, when employed as a cathode material in LIBs, exhibited a good charge/discharge capacity of 151/128 mAh g^–1^ and stable cyclability.^30^ Although LIBs remain the dominant technology for portable electronic devices, the scarcity and high cost of lithium resources have prompted efforts to develop alternative battery systems based on more abundant elements. To this end, Lan and co-workers employed a CoPc-O-Mn MPc-based MOF in a light-assisted Li–CO_2_ battery device, leveraging features of the MOF including the dual active metal-sites, high conductivity, and photosensitivity (Figure 8). The resulting battery exhibited a high round-trip efficiency of 98.5%, an ultralow voltage hysteresis of 0.05 V, and excellent cycling-stability (81.3%) for 60 h at a current density of 0.02 mA cm^–2^.^45^

**Figure 8:**
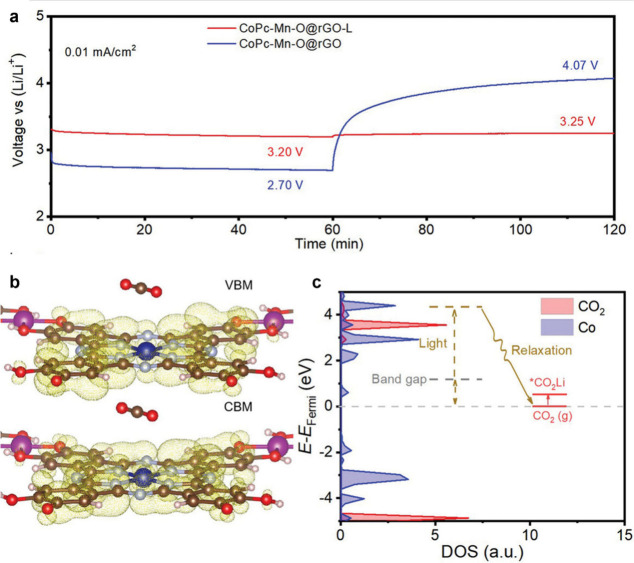
(a) Discharge and charge curves of the CoPc-O-Mn@rGO based Li–CO_2_ battery with and without illumination at 0.01 mA cm^–2^. (b) Projected charge density of the valence band maximum (VBM) and conduction band minimum (CBM) of CoPc-O-Mn. (c) Density states of CoPc-O-Mn; red line denotes CO_2_, and blue line denotes Co. Reproduced with permission from reference 45. Copyright 2022 Wiley-VCH.

In summary, conductive MPc-based MOFs have demonstrated promising applicability in the context of energy storage devices, enabling high-performance capacitors and advanced battery systems. The modular structures, intrinsic conductivity, and redox-active sites of these materials collectively contribute to enhanced charge storage, stability, and versatility across both aqueous and nonaqueous systems. Future studies may look to investigate the role of guest molecule diffusion to interlayer active sites within energy storage applications.

### 3.4 Magnetic Device Applications for MPc-Based MOFs

MPc molecules are well-known for possessing magnetic properties, owing to the metal ion within the core of the phthalocyanine.^48,49^ The magnetism related capabilities of MPc-based MOFs is of great interest, as theoretical studies have highlighted the promising magnetic potential of these materials.^19^ For example, in study by Li and co-workers, DFT calculations showed that a manganese containing MPc-based MOF, MnPc-NH-Ni, displayed room temperature ferromagnetism resulting from the uniquely strong hybridization between the d-π orbitals of Mn, the Pc ring, and the Ni bis­(diimine) nodes.^47^

The theorized magnetic potential of MPc-based MOFs was realized in a study by Feng and co-workers.^24^ They synthesized and characterized an iron containing MOF, K_3_[FePc-O-Fe], which displayed spontaneous magnetization properties including long-range magnetic correlations within the framework material.^24^ By using time-resolved Tetrahertz spectroscopy in conjunction with DFT calculations, the authors determined that the superparamagnetic nature of K_3_ [FePc-O-Fe] at 350 K results from the strong hybridization between the d-p orbitals of both M_1_ and M_2_ iron species.^24^ This magnetic coupling is further supported in a separate study in which Dong and co-workers noted that MPc-based MOFs may demonstrate interlayer ferromagnetic coupling as evidenced by the decrease in interlayer stacking distance between MOFs with CuPc cores and MOFs with FePc cores.^28^

Further leveraging the magnetic potential of MPc-based MOFs, Hu and co-workers applied MPc-based MOFs in spintronic devices using MPc-O-Cu (M = Ni, Cu, H_2_) MOF thin films (Figure 9).^31^ In this study, the researchers assembled the thin films via a programmed layer-by-layer approach within a vertical configuration wherein the MPc-based MOF layer acted as a spin valve. The devices demonstrated notably high negative magnetoresistance (MR) of –22% at 50 K.^31^ This study showed that cobalt atoms present within the device layers coordinated with unreacted catecholate edge sites of the MOFs, resulting in an antiferromagnetic layer of the MPc-O-Cu/Co hybrid structure.^31^

**Figure 9:**
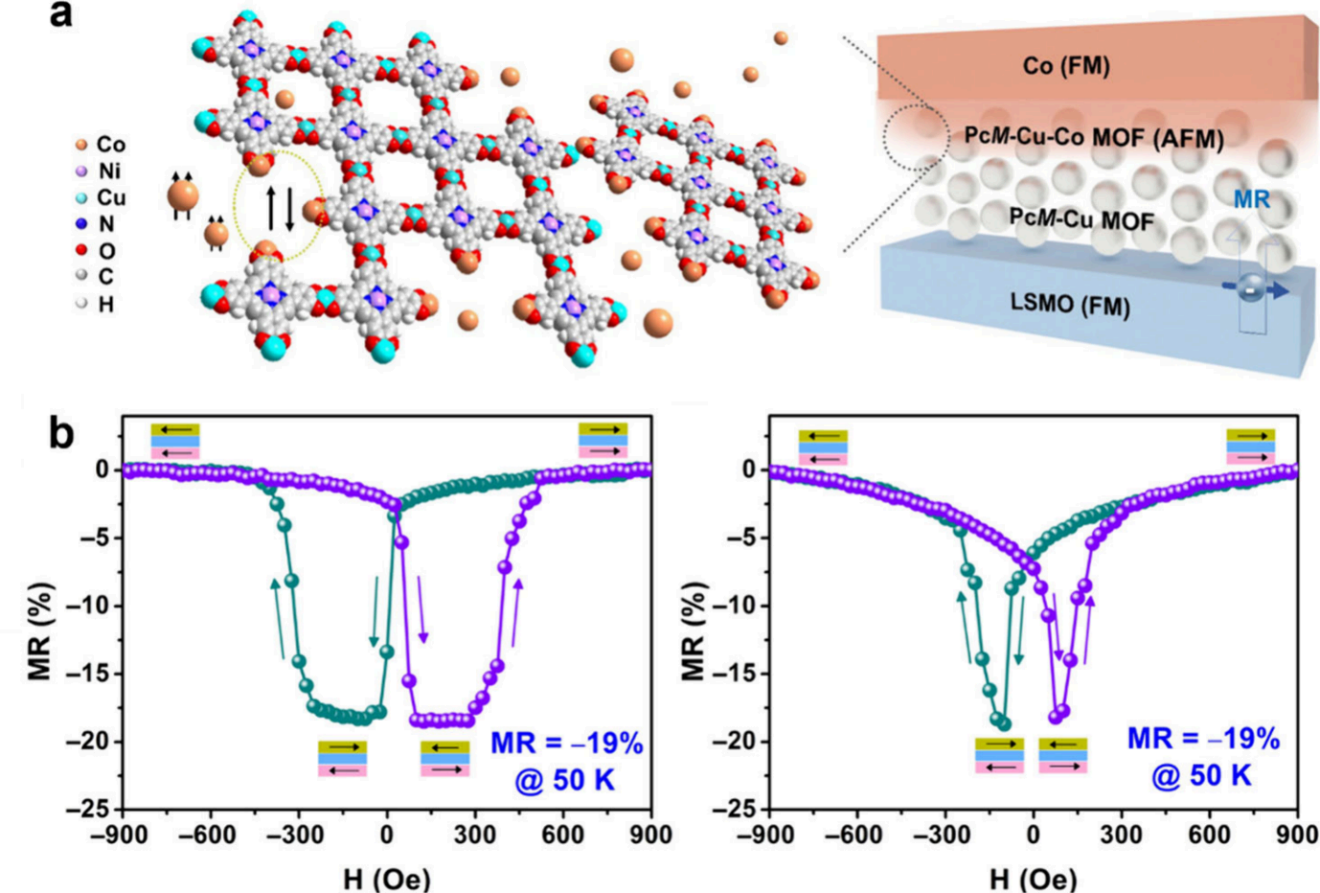
NiPc-O-Cu MOF-based organic spin valves (OSVs), and possible ferromagnetic-antiferromagnetic (FM-AFM) coupling mechanism. (b) MR and M−H measurements. MR loops for (left) LSMO/NiPc-O-Cu-30 °C (∼85 nm)/Co/Au and (right) LSMO/CuPc-O-Cu-30C (∼92 nm)/Co/Au measured at *T* = 50K, I = 0.1 μA. Reproduced with permission from reference 31. Copyright 2024 Chinese Chemical Society.

In summary, research efforts have both predicted the room temperature magnetic ordering of MPc-based MOFs with DFT level calculations and have experimentally demonstrated the magnetic utility of MPc-based MOFs within a magnetoresistive spintronic device. While MPc-based MOFs show great promise within the field of magnetism related applications, more fundamental insight is required to enhance the understanding of how both structural and metal ion modularity impact the magnetic properties of MPc-based MOFs.

## 4 Conclusions and Outlook

In this Account, we summarize the current state of the field of MPc-based MOFs within functional devices, with a focus on how the structure of the MPc-based MOF dictates the function of the material. We highlight studies that survey the unique relationship between MPc-based MOF structure and the emergent impact on the physicochemical properties of the materials. Furthermore, we discuss how these structure–property relationships of the MPc-based MOFs enhance the functionality of these materials within applications, such as chemical sensing, electrocatalysis, energy storage, and magnetism related applications.

Despite these advances, there are at least three primary challenges that must be addressed both to codify the understanding of the structure–property relationships of MPc-based MOFs and to ensure the continued development of this field. *First*, solving the crystal structure of an MPc-based MOF is essential to establish a reliable structural foundation, which in turn is critical for the long-term development of their electronic applications. At present, structural understanding largely relies on comparisons between experimental PXRD traces and simulated PXRD patterns, supplemented by TEM imaging and FFT diffraction analysis. While these methods provide insight into the in-plane crystallinity of MPc-based MOFs, fundamental questions remain about the degree of long-range 2D order and the nature of interlayer stacking mode. The stacking arrangements of the interlayers holds major structure–property implications for MPc-based MOFs for two reasons: (**1**) interlayer arrangement will dictate the accessibility of guest molecules for MPc-based MOFs within both electrochemical and energy storage applications; and (**2**) interlayer arrangement will critically affect the electronic properties of MPc-based MOFs, since variations in eclipsed versus inclined stacking directly modulate charge-transport pathways.^23^ Thus, validating the precise arrangement and stacking modes of MPc-based MOFs in two dimensions through advanced characterizations techniques, such as single-crystal XRD, pair and radial distribution function analysis, atomic-resolution TEM/scanning TEM, and microcrystal electron diffraction (MicroED), will be indispensable for guiding both fundamental studies and practical device integration. The use of these strategies has already proven fruitful for the investigation of other layered framework materials,^50,51^ and can, in principle, be extended to MPc-based MOFs.

*Second*, we, as researchers, must improve the accessibility of MPc-based MOF synthesis and the repeatability of MPc-based MOF reports. Synthetic procedures for both MPc monomers and MPc-based MOFs should not be merely referenced, but should be described in full detail by the authors, with key visual information and characterization data, including NMR, elemental analysis, PXRD, and SEM, provided whenever applicable, to ensure batch-to-batch reproducibility and cross-laboratory validation. With standardized synthetic procedures established for MPc-based MOFs, materials properties such as crystallinity, 4-point probe conductivity, and BET surface area will likely stabilize from report to report. As materials properties stabilize for MPc-based MOFs, the field has an opportunity to conduct critical investigations regarding structural parameters of MPc-based MOFs (e.g., degree of crystallinity, pore structure, impact of size on material performance) to generate molecular engineering level insight into the porosity of MPc-based MOFs. Before MPc-based MOFs can be reliably harnessed in applications and devices, agreed upon standards for material characterization and materials properties are needed to ensure that future reporting is built from a robust knowledge base, as suggested by recently proposed guidelines for MOF reporting.^52^

*Third*, it is imperative that the processability and device integration strategies of MPc-based MOFs are improved to widen the breadth of applications for MPc-based MOF utilization. While the field of MPc-based MOFs is still developing, the field of MPc molecules is well established, and MPcs are well-known for their utility within a wide range of applications. MPc-based MOFs are hindered from ubiquitous use within the same applications as MPcs because the MOFs are insoluble and thus are less facile for device integration. Efforts to broaden the processability of MPc-based MOFs potentially including ball-milled polymeric mixes,^53^ composites,^54^ thin film *in situ* deposition and growth methods,^55^ and self-assembly onto textiles^56^ are necessary to widen the scope of MPc-based MOF applications.

Concerted efforts addressing these challenges are crucial to enhance the field of MPc-based MOFs from a material discovery perspective and to broaden the applicability of MPc-based MOFs. MPc-based MOFs offer unique attributes including a Lieb lattice structure and the intrinsic bimetallic system, arising from the metal centers in both MPc core and the metal nodes, along with 2D MOF materials properties, such as inherent porosity, structural and molecular modularity, and electrical conductivity which results in a designer framework material capable of emergent functionality greater than the sum of its parts. The research into these materials is at a critical juncture, where future applications may be highly impactful, but challenges in scaling up material synthesis, as well as materials processability limit ubiquitous utilization. It is up to us as materials chemists to help bridge these gaps and iteratively work toward a future, where MPc-based MOFs are a viable material for next-generation applications.

## Supplementary Material



## Notes

Table summarizing first reports and applications of each organic linker within 2D MOFs (PDF)
