# Supplementary material for: Framing Function: Metallophthalocyanine-Based Metal–Organic Frameworks as Multifunctional Materials for Electrified Devices
Source: Acc Mater Res. 2026 Jan 13;7(2):165–78. doi: 10.1021/accountsmr.5c00283 (PMC12954758; doi:10.1021/accountsmr.5c00283)
Supplement: Supplementary file 1 [file mr5c00283_si_001.pdf]

Supporting Information

Framing Function: Metallophthalocyanine-based Metal–Organic Frameworks as Multifunctional Materials for Electrified Devices

Authors: Evan L. Cline†, Hyuk-Jun Noh†, and Katherine A. Mirica*

Burke Laboratory, Dartmouth College, 41 College Street, Hanover, New Hampshire 03755, United States. Email: Katherine.A.Mirica@dartmouth.edu

† These authors contributed equally

* Correspondence: Katherine.A.Mirica@dartmouth.edu

Linker skeleton	Heteroatomic linker atom	Metal Node(s) used	Application within first report of MOF
**Triphenylene (HXTP)**	O	Cu, Ni, Co	Energy Storage [1]
	NH	Ni	Charge Transport Properties [2]
	SH	Pt	Ion Uptake[3]
	SeH	Co	Magnetism[4]
**Benzene (HXB)**	S	Ni	Charge Transport Properties [5]
	NH	Cu, Ni	Charge Transport Properties[6]
	O	Cu	Charge Transport Properties [7]
	SeH	Cu	Charge Transport Properties[8]
**Coronene (PXC)**	S	Fe	Ferromagnetism [9]
**Metallophthalocyanine (MPc)**	O	Cu	Lithium Battery[10]
	NH	Cu	Catalysis [11]
	S	Ni	Capacitor[12]
**Metallonaphthalocyanine (MNPc)**	O	Ni, Cu	Chemiresistive sensing [13]
**Trinaphthylene (HXTN)**	O	Cu	Charge Transport Properties [14]
**Dibenzo-[g,p]chrysene (DBC)**	O	Cu	Energy Storage [15]
**Truxene**	O	Cu	Electrochemical Sensing [16]
**Hexaazatrinaphthylene (HATNA)**	O	Cu	Catalysis [17]
**Tetraazanaphthotetraphene (HXTT)**	O	Cu, Ni, Co, Mg	Electronic Transport Properties[18]
**Triindole (HXTI)**	NH	Ni	Thermoelectric Properties [19]
**Tribenzocyclyne (HHTC)**	O	Cu	Charge Transport Properties [20]
**Benzocoranene (HBC)**	O	Cu	Charge Transport Properties [21]
**Cu-Salphen**	O	Cu	Chemiresistive Sensing [22]
**Tribenzoimidazole (HXTBim)**	NH	Ni	Electronic Transport Properties[23]
**Triphenylenotrisbenzodioxin (HAOTP)**	NH	Ni, Cu	Chemiresistive Sensing [24]
**(TBA) Tetrabenzoanthracene**	O	Cu	Energy Storage[25]
**Ethynylphenanthrene (EP)**	O	Cu	Ion Uptake [26]
**Triaminotriphenylene (HHTAP)**	O	Cu	Proton Conductivity [27]
**ThiaTRX**	O	Cu	Proton Conductivity [28]

Table S1 Summarizing first reports and applications of each organic linker and 2D MOF material.

References

(1) Hmadeh, M.; Lu, Z.; Liu, Z.; Gándara, F.; Furukawa, H.; Wan, S.; Augustyn, V.; Chang, R.; Liao, L.; Zhou, F.; Perre, E.; Ozolins, V.; Suenaga, K.; Duan, X.; Dunn, B.; Yamamto, Y.; Terasaki, O.; Yaghi, O. M. New Porous Crystals of Extended Metal-Catecholates. *Chemistry of Materials* **2012**, *24* (18), 3511–3513. https://doi.org/10.1021/cm301194a.

(2) Sheberla, D.; Sun, L.; Blood-Forsythe, M. A.; Er, S.; Wade, C. R.; Brozek, C. K.; Aspuru-Guzik, A.; Dincă, M. High Electrical Conductivity in Ni3(2,3,6,7,10,11- Hexaiminotriphenylene)2, a Semiconducting Metal-Organic Graphene Analogue. *J Am Chem Soc* **2014**, *136* (25), 8859–8862. https://doi.org/10.1021/ja502765n.

(3) Cui, J.; Xu, Z. An Electroactive Porous Network from Covalent Metal-Dithiolene Links. *Chemical Communications* **2014**, *50* (30), 3986–3988. https://doi.org/10.1039/c4cc00408f.

(4) Cui, Y.; Yan, J.; Chen, Z.; Xing, W.; Ye, C.; Li, X.; Zou, Y.; Sun, Y.; Liu, C.; Xu, W.; Zhu, D. Synthetic Route to a Triphenylenehexaselenol-Based Metal Organic Framework with Semi-Conductive and Glassy Magnetic Properties. *iScience* **2020**, *23* (1), 100812. https://doi.org/10.1016/j.isci.2019.100812.

(5) Kambe, T.; Sakamoto, R.; Hoshiko, K.; Takada, K.; Miyachi, M.; Ryu, J. H.; Sasaki, S.; Kim, J.; Nakazato, K.; Takata, M.; Nishihara, H. π-Conjugated Nickel Bis(Dithiolene) Complex Nanosheet. *J Am Chem Soc* **2013**, *135* (7), 2462–2465. https://doi.org/10.1021/ja312380b.

(6) Dou, J. H.; Sun, L.; Ge, Y.; Li, W.; Hendon, C. H.; Li, J.; Gul, S.; Yano, J.; Stach, E. A.; Dincă, M. Signature of Metallic Behavior in the Metal-Organic Frameworks M3(Hexaiminobenzene)2 (M = Ni, Cu). *J Am Chem Soc* **2017**, *139* (39), 13608–13611. https://doi.org/10.1021/jacs.7b07234.

(7) Park, J.; Hinckley, A. C.; Huang, Z.; Feng, D.; Yakovenko, A. A.; Lee, M.; Chen, S.; Zou, X.; Bao, Z. Synthetic Routes for a 2D Semiconductive Copper Hexahydroxybenzene Metal-Organic Framework. *J Am Chem Soc* **2018**, *140* (44), 14533–14537. https://doi.org/10.1021/jacs.8b06666.

(8) Cui, Y.; Yan, J.; Chen, Z.; Zhang, J.; Zou, Y.; Sun, Y.; Xu, W.; Zhu, D. [Cu 3 (C 6 Se 6)] N : The First Highly Conductive 2D π–d Conjugated Coordination Polymer Based on Benzenehexaselenolate. *Advanced Science* **2019**, *6* (9). https://doi.org/10.1002/advs.201802235.

(9) Dong, R.; Zhang, Z.; Tranca, D. C.; Zhou, S.; Wang, M.; Adler, P.; Liao, Z.; Liu, F.; Sun, Y.; Shi, W.; Zhang, Z.; Zschech, E.; Mannsfeld, S. C. B.; Felser, C.; Feng, X. A Coronene-Based Semiconducting Two-Dimensional Metal-Organic Framework with Ferromagnetic Behavior. *Nat Commun* **2018**, *9* (1). https://doi.org/10.1038/s41467-018-05141-4.

(10) Nagatomi, H.; Yanai, N.; Yamada, T.; Shiraishi, K.; Kimizuka, N. Synthesis and Electric Properties of a Two-Dimensional Metal-Organic Framework Based on Phthalocyanine. *Chemistry – A European Journal* **2018**, *24* (8), 1806–1810. https://doi.org/10.1002/chem.201705530.

(11) Jia, H.; Yao, Y.; Zhao, J.; Gao, Y.; Luo, Z.; Du, P. A Novel Two-Dimensional Nickel Phthalocyanine-Based Metal–Organic Framework for Highly Efficient Water Oxidation Catalysis. *J Mater Chem A Mater* **2018**, *6* (3), 1188–1195. https://doi.org/10.1039/C7TA07978H.

(12) Zhang, P.; Wang, M.; Liu, Y.; Fu, Y.; Gao, M.; Wang, G.; Wang, F.; Wang, Z.; Chen, G.; Yang, S.; Liu, Y.; Dong, R.; Yu, M.; Lu, X.; Feng, X. Largely Pseudocapacitive Two-Dimensional Conjugated Metal–Organic Framework Anodes with Lowest Unoccupied Molecular Orbital

Localized in Nickel-Bis(Dithiolene) Linkages. *J Am Chem Soc* **2023**, *145* (11), 6247–6256. https://doi.org/10.1021/jacs.2c12684.

(13) Meng, Z.; Aykanat, A.; Mirica, K. A. Welding Metallophthalocyanines into Bimetallic Molecular Meshes for Ultrasensitive, Low-Power Chemiresistive Detection of Gases. *J Am Chem Soc* **2019**, *141* (5), 2046–2053. https://doi.org/10.1021/jacs.8b11257.

(14) Meng, Z.; Mirica, K. A. Two-Dimensional d-π Conjugated Metal-Organic Framework Based on Hexahydroxytrinaphthylene. *Nano Res* **2021**, *14* (2), 369–375. https://doi.org/10.1007/s12274-020-2874-x.

(15) Liu, J.; Zhou, Y.; Xie, Z.; Li, Y.; Liu, Y.; Sun, J.; Ma, Y.; Terasaki, O.; Chen, L. Conjugated Copper–Catecholate Framework Electrodes for Efficient Energy Storage. *Angewandte Chemie* **2020**, *132* (3), 1097–1102. https://doi.org/10.1002/ange.201912642.

(16) Zhao, Q.; Li, S. H.; Chai, R. L.; Ren, X.; Zhang, C. Two-Dimensional Conductive Metal-Organic Frameworks Based on Truxene. *ACS Appl Mater Interfaces* **2020**, *12* (6), 7504–7509. https://doi.org/10.1021/acsami.9b23416.

(17) Liu, Y.; Li, S.; Dai, L.; Li, J.; Lv, J.; Zhu, Z.; Yin, A.; Li, P.; Wang, B. The Synthesis of Hexaazatrinaphthylene-Based 2D Conjugated Copper Metal-Organic Framework for Highly Selective and Stable Electroreduction of CO2 to Methane. *Angewandte Chemie - International Edition* **2021**, *60* (30), 16409–16415. https://doi.org/10.1002/anie.202105966.

(18) Dou, J. H.; Arguilla, M. Q.; Luo, Y.; Li, J.; Zhang, W.; Sun, L.; Mancuso, J. L.; Yang, L.; Chen, T.; Parent, L. R.; Skorupskii, G.; Libretto, N. J.; Sun, C.; Yang, M. C.; Dip, P. V.; Brignole, E. J.; Miller, J. T.; Kong, J.; Hendon, C. H.; Sun, J.; Dincă, M. Atomically Precise Single-Crystal Structures of Electrically Conducting 2D Metal–Organic Frameworks. *Nat Mater* **2021**, *20* (2), 222–228. https://doi.org/10.1038/s41563-020-00847-7.

(19) Lu, Y.; Zhang, Y.; Yang, C. Y.; Revuelta, S.; Qi, H.; Huang, C.; Jin, W.; Li, Z.; Vega-Mayoral, V.; Liu, Y.; Huang, X.; Pohl, D.; Položij, M.; Zhou, S.; Cánovas, E.; Heine, T.; Fabiano, S.; Feng, X.; Dong, R. Precise Tuning of Interlayer Electronic Coupling in Layered Conductive Metal-Organic Frameworks. *Nat Commun* **2022**, *13* (1). https://doi.org/10.1038/s41467-022-34820-6.

(20) Pham, H. T. B.; Choi, J. Y.; Huang, S.; Wang, X.; Claman, A.; Stodolka, M.; Yazdi, S.; Sharma, S.; Zhang, W.; Park, J. Imparting Functionality and Enhanced Surface Area to a 2D Electrically Conductive MOF via Macrocyclic Linker. *J Am Chem Soc* **2022**, *144* (23), 10615–10621. https://doi.org/10.1021/jacs.2c03793.

(21) Xing, G.; Liu, J.; Zhou, Y.; Fu, S.; Zheng, J. J.; Su, X.; Gao, X.; Terasaki, O.; Bonn, M.; Wang, H. I.; Chen, L. Conjugated Nonplanar Copper-Catecholate Conductive Metal-Organic Frameworks via Contorted Hexabenzocoronene Ligands for Electrical Conduction. *J Am Chem Soc* **2023**, *145* (16), 8979–8987. https://doi.org/10.1021/jacs.2c13835.

(22) Su, X.; Zhong, Z.; Yan, X.; Zhang, T.; Wang, C.; Wang, Y.; Xu, G.; Chen, L. Facile Synthesis of Metallosalphen-Based 2D Conductive Metal-Organic Frameworks for NO_2 Sensing: Metal Coordination Induced Planarization. *Angewandte Chemie International Edition* **2023**, *62* (22). https://doi.org/10.1002/anie.202302645.

(23) Apostol, P.; Gali, S. M.; Su, A.; Tie, D.; Zhang, Y.; Pal, S.; Lin, X.; Bakuru, V. R.; Rambabu, D.; Beljonne, D.; Dincă, M.; Vlad, A. Controlling Charge Transport in 2D Conductive MOFs—The

Role of Nitrogen-Rich Ligands and Chemical Functionality. *J Am Chem Soc* **2023**. https://doi.org/10.1021/jacs.3c07503.

(24) Chen, P.; Su, X.; Wang, C.; Zhang, G.; Zhang, T.; Xu, G.; Chen, L. Two-Dimensional Conjugated Metal-Organic Frameworks with Large Pore Apertures and High Surface Areas for NO2 Selective Chemiresistive Sensing. *Angewandte Chemie - International Edition* **2023**, *62* (40). https://doi.org/10.1002/anie.202306224.

(25) Cheng, L.; Qi, M.; Yu, J.; Zhang, X.; Wang, H. G.; Cui, F.; Wang, Y. Conjugation and Topology Engineering of 2D π-d Conjugated Metal–Organic Frameworks for Robust Potassium Organic Batteries. *Angewandte Chemie - International Edition* **2024**, *63* (25), e202405239. https://doi.org/10.1002/anie.202405239.

(26) Pham, H. T. B.; Choi, J. Y.; Fang, X.; Claman, A.; Huang, S.; Coates, S.; Wayment, L.; Zhang, W.; Park, J. Macrocyclic Ligand-Driven Ion Selectivity and High Surface Area in a 2D Conductive MOF. *Chem* **2024**, *10* (1), 199–210. https://doi.org/10.1016/j.chempr.2023.08.026.

(27) Park, G.; Demuth, M. C.; Hendon, C. H.; Park, S. S. Acid-Dependent Charge Transport in a Solution-Processed 2D Conductive Metal-Organic Framework. *J Am Chem Soc* **2024**, *146* (16), 11493–11499. https://doi.org/10.1021/jacs.4c02326.

(28) Lu, C.; Choi, J. Y.; Check, B.; Fang, X.; Spotts, S.; Nuñez, D.; Park, J. Thiatruxene-Based Conductive MOF: Harnessing Sulfur Chemistry for Enhanced Proton Transport. *J Am Chem Soc* **2024**, *146* (38), 26313–26319. https://doi.org/10.1021/jacs.4c08659.
